# Double arc aortique: à propos de deux cas

**DOI:** 10.11604/pamj.2017.27.273.13481

**Published:** 2017-08-10

**Authors:** Sihame Lemouakni, Amale Hassani, Hakim Elyajouri, Mohammed Kmari, Hakim Ourrai, Rachid Abilkacem, Aomar Agadr

**Affiliations:** 1 Service de Pédiatrie, Hôpital Militaire Mohamed V, Rabat, Maroc

**Keywords:** Double arc aortique, anneau vasculaire, Stridor, Double aortic arch, vascular ring, stridor

## Abstract

Le double arc aortique représente une anomalie rare de l’arc aortique. Il provient de l’absence d’involution de l’aorte dorsale caudale. La symptomatologie clinique est habituellement précoce, notée dès la période néonatale ou peu après, dominée par des signes respiratoires et digestives. Le TOGD permet un diagnostic précis de l’anomalie. Cependant, l’angiographie est d’un grand intérêt diagnostique ainsi que dans le choix de l’approche thérapeutique. Seul le traitement chirurgical permet de lever les signes compressifs sur l’axe trachéo-oesophagien. L’objectif de notre travail est d’illustrer à travers deux observations de double aortique chez deux patients âgés respectivement de 7 mois et 9 mois, l’apport de l’imagerie dans le diagnostic difficile de cette anomalie.

## Abstract (en)

Double aortic arch is a rare anomaly of the aortic arch. It is due to the absence of involution of the caudal dorsal aorta. The disease usually begins to show itself in very early clinical signs, already detectable in the neonatal period. Angiography is of great interest to its diagnosis as well as to the choice of the therapeutic approach. Only surgical treatment allows to eliminate tracheoesophageal compression. Surgical mortality rate is low thanks to the progress of postoperative resuscitation. We here report two cases of double aortic arch in order to highlight the contribution of imaging in the difficult diagnosis of this anomaly.

## Introduction

Le double arc aortique est une anomalie rare de l’arc aortique. Il provient de l’absence d’involution de l’aorte dorsale caudale [**1**]. La symptomatologie clinique est habituellement précoce, notée dès la période néonatale. L’angiographie est d’un grand intérêt diagnostique ainsi que dans le choix de l’approche thérapeutique. Seul le traitement chirurgical permet de lever les signes compressifs sur l’axe trachéo-œsophagien. La mortalité opératoire est devenue faible grâce aux progrès de la réanimation postopératoire. Nous rapportons à travers deux observations de double arc aortique, l’apport de l’imagerie dans le diagnostic difficile de cette anomalie.

## Patient et observation

### Observation 1

**Figure 1:**
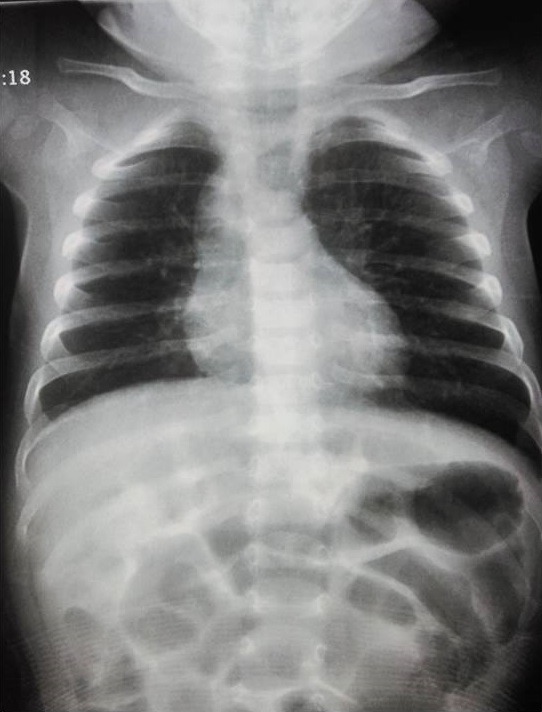
Radiographie du thorax montrant une distension thoracique avec effacement du bouton aortique

**Figure 2:**
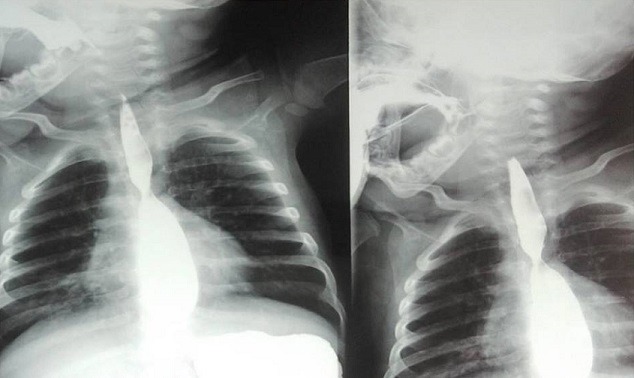
Image d’empreinte extrinsèque au TOGD

**Figure 3:**
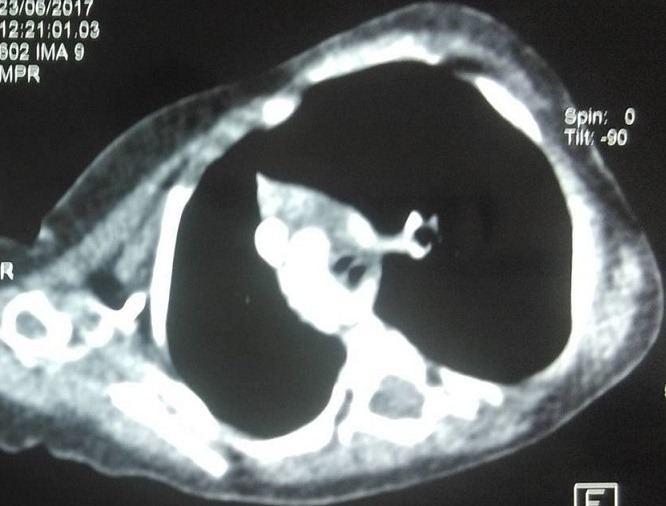
Coupe scanographique axiale montrant un Double arc aortique encerclant l’axe oesotrachéal

**Figure 4:**
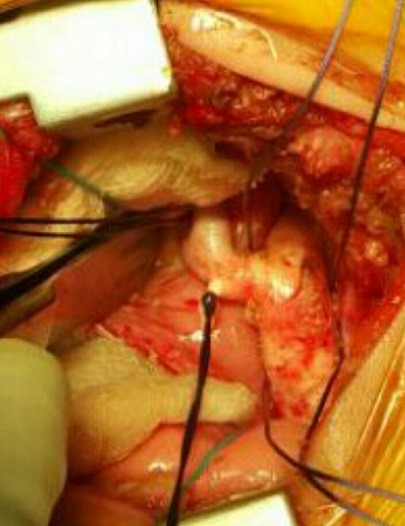
Vue préopératoire d’un double arc aortique encerclant l’œsophage

**Figure 5:**
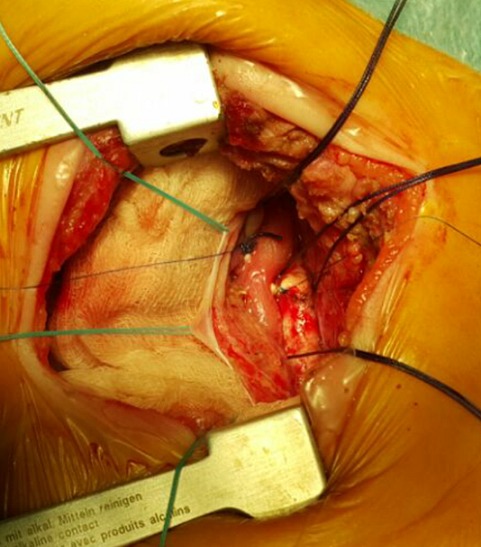
Vue préopératoire montrant la section de l’arc aortique gauche

Nourrisson de sexe féminin, âgée de 7 mois, issue d’une grossesse mal suivie menée à terme, accouchement par voie basse à domicile, cris immédiat, consanguinité 1ere degré. Ayant comme antécédent notion d’hospitalisation à J2O de vie pour BAV 1 ère épisode. Admis dans notre service pour prise en charge d’une dyspnée avec stridor en deux temps. Chez qui examen clinique trouve un nourrisson polypnéique, fébrile, des râles sibilants à l’auscultation, avec des signes de lutte respiratoire. Une radiographie du thorax a objectivé une distension thoracique, absence de bouton aortique avec un index cardio-thoracique à 0,5 (**Figure 1**). Le transit oeso-gastro-duodénale a montré une empreinte anormale sur œsophage (**Figure 2**). Le bilan paraclinique a été complété par un angioscanner thoracique objectivant un double arc aortique, un foyer de condensation du lobe moyen d’allure infectieuse (**Figure 3**) avec une échographie cardiaque normale. Elle a bénéficiée au cours du geste opératoire d’une section suture de l’arc aortique gauche qui était en avant de l’œsophage avec section du ligament artériel et libération du tractus oesotrachéale (**Figure 4**, **Figure 5**). Evolution a été marquée par installation d’un choc septique et la patiente est décédée à J7 de post opératoire.

### Observation 2

**Figure 6:**
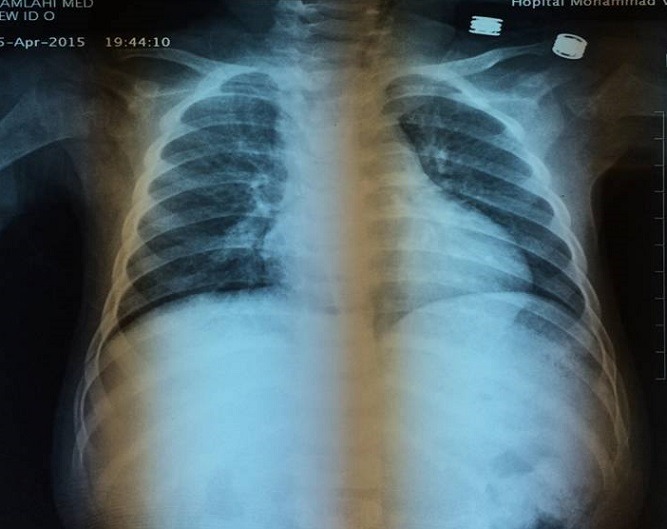
Radiographie thoracique montrant une déviation trachéale à gauche avec un index cardiothoracique normale

**Figure 7:**
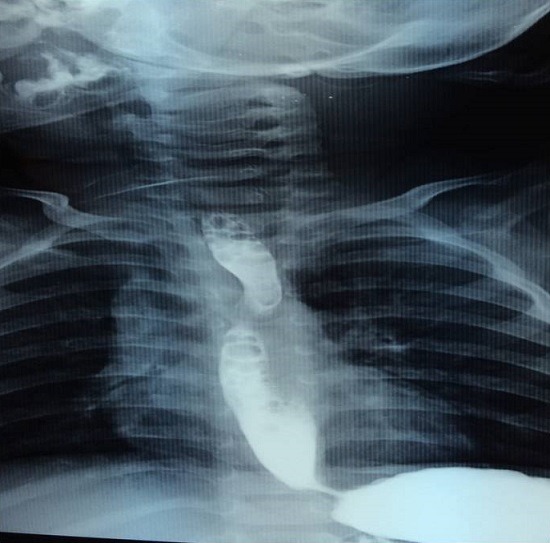
Transit oeso-gastro-duodénale montrant une empreinte sur œsophage

Nourrisson de sexe masculin, âgée de 9 mois, accouchement par voie basse à terme, pas de notion de consanguinité, qui présente depuis l’âge de 1 mois stridor, encombrement bronchique avec notion de trois épisodes de dyspnées sifflantes. Le malade a été mis sous corticothérapie inhalée 500μg/j depuis 2 mois mais sans amélioration. Devant la persistance d’un wheezing le nourrisson a été admis dans notre formation pour prise en charge où une radiographie thoracique a été réalisée montrant une déviation trachéale à gauche, absence de bouton aortique avec un index cardiothoracique normale (**Figure 6**). Le transit oeso-gastro-duodénale a objectivé une empreinte sur œsophage (**Figure 7**). La Fibroscopie bronchique a montré une Compression battante de l’extrémité inférieure de la trachée. L’angioscanner thoracique a confirmé le diagnostic d’un double arc aortique. Et le malade a été adressé en chirurgie pour complément de prise en charge.

## Discussion

Le double arc aortique est dû à la persistance de la partie distale de l’aorte dorsale droite, entrainant la formation de crosses aortiques droite et gauche qui entourent la trachée et l’œsophage avant de se rejoindre pour former l’aorte thoracique descendante. Chaque crosse fournit l’origine de l’artère carotide primitive et de l’artère sous-clavière homolatérale. Habituellement, les deux crosses sont perméables, auquel cas la droite ou la gauche peut être dominante ou elles peuvent être de taille égale. Dans 75% des cas la crosse droite est dominante selon la littérature [**2****–****5**] comme étant le cas de nos patients. L´âge de révélation de la maladie est précoce chez nos deux malades (7 mois, 9 mois), ce qui semble concordant avec la littérature qui est en général avant 3 ans [**1**], bien que dans 25% des cas, le diagnostic est réalisé à un âge adulte [**6**]. Le double arc aortique peut être difficile à diagnostiquer car les symptômes ne sont pas typiques d’un trouble cardiaque. Le tableau clinique est Dominé par des symptômes respiratoires (stridor, détresse respiratoire, toux chronique) et digestives (dysphagie) [**7**, **8**]. L’importance des signes dépend de l’espace entre les deux arcs aortiques. Parfois le diagnostic peut être confondu avec un asthme, une bronchiolite ou pneumonies à répétition. C ‘est le cas de notre patient qui a été traité comme asthme avec une corticothérapie inhalée a forte dose mais sans amélioration.

Cette anomalie vasculaire congénitale peut été isolée ou associée des malformations cardiaques comme la tétralogie de Fallot, communication interventriculaire, persistance du canal artériel, atrésie pulmonaire à septum ouvert, coarctation de l’aorte Ou à des malformations extracardiaques comme la microdélétion du chromosome 22q11 dans le syndrome de Di George. Echographie cardiaque de nos patients est revenue normale. L’imagerie occupe une place prépondérante dans le diagnostic du double arc aortique. La radiographie thoracique est examen de première intention. Elle permet parfois à elle seule d’évoquer le diagnostic de compression d’origine vasculaire .La fibroscopie permet de préciser le degré de compression et d’éliminer les diagnostics différentiels. Le transit oesogastroduodénale permet parfois en fonction du siège et de l’orientation de l’empreinte de préciser le type d’anomalie. Tout enfant symptomatique dont le TOGD met en évidence une empreinte œsophagienne anormale doit bénéficier d’une imagerie en coupes. Angioscanner est l’examen de référence, elle permet de faire le diagnostic, de préciser son type et ses rapports avec les structures adjacentes. Actuellement grâce à la nouvelle technologie, un angioscanner avec reconstruction donne d’excellente image en deux ou trois dimensions [**9**, **10**]. IRM permet comme le scanner de faire un bilan anatomique précis, elle n’est pas irradiante et réputée comme non invasive, cependant elle nécessite une sédation lourde. Le traitement est exclusivement chirurgical [**10**–**12**]. L’indication du geste opératoire est indiquée si le syndrome de compression œsotrachéale est sévère avec des infections respiratoires à répétition et des épisodes d’asphyxie qui peuvent se compliquer par un arrêt cardiorespiratoire. La thoracotomie postéro-latérale gauche est la voie de référence pour les différents auteurs. Une équipe chirurgicale expérimentée est nécessaire avec une bonne préparation anesthésique.

## Conclusion

Le double arc aortique, reste une pathologie assez rare qu’il faut y penser devant une Détresse respiratoire néonatale sans étiologie évidente, devant un Asthme sévère du nourrisson ou bronchiolite à répétition. Le traitement chirurgical est indiqué si la symptomatologie est franche et/ou mise en jeu du pronostic vital. Généralement l’évolution postopératoire est satisfaisante. Un caryotype est à demandé à la recherche micro délétion 22q11.

## Conflits d’intérêts

Les auteurs ne déclarent aucun conflit d'intérêts.
